# Water sorption isotherms and mid-infrared spectra of dried parchment coffee beans (*Coffee arabica* L.) processed by wet and semi-dry postharvest methods. A dataset for estimating water sorption and thermodynamic properties.

**DOI:** 10.1016/j.dib.2024.110958

**Published:** 2024-09-19

**Authors:** Gentil A. Collazos-Escobar, Valeria Hurtado-Cortés, Andrés F. Bahamón-Monje, Nelson Gutiérrez-Guzmán

**Affiliations:** a Grupo de Análisis y Simulación de Procesos Agroalimentarios (ASPA), Instituto Universitario de Ingeniería de Alimentos–FoodUPV, Universitat Politècnica de València, Camí de Vera s/n, Edificio 3F, 46022 Valencia, Spain; b Centro Surcolombiano de Investigación en Café (CESURCAFÉ), Departamento de Ingeniería Agrícola, Universidad Surcolombiana, Neiva-Huila, Colombia

**Keywords:** Hygroscopicity, Equilibrium moisture content, Differential/integral sorption properties, Mathematical modelling

## Abstract

This work contains a water sorption isotherms dataset obtained on dried parchment coffee beans processed by wet and semi-dry postharvest methods and their mid-infrared spectral data. The experimental data of water sorption isotherms were determined using the Dynamic Dewpoint Isotherm (DDI) method. The measurements were taken in a water activity range of 0.1 to 0.85 and at 25, 35, and 45 °C temperatures. To spectrally characterize the dried parchment coffee beans processed by wet and semi-dry postharvest methods, the Attenuated Total Reflection Fourier Transform Infrared (ATR-FTIR) spectroscopy technique was used. The dataset comprises Excel files with the experimental data acquired for the dried parchment coffee beans processed by wet and semi-dry postharvest methods and the experimental conditions assessed. This dataset serves as a reliable and valuable tool for researchers, coffee producers, and decision-makers to be used as the basis for mathematically computing relevant parameters related to the coffee shelf life and hygroscopic behavior, as well as to develop suitable packaging materials/containers to maximize the quality of coffee beans in terms of sensory flavors and moisture stability. Furthermore, the experimental data provide a reliable tool for optimizing the coffee storage process and gaining insights into the water-sorption process.

Specifications Table

**Table:** 

Subject	Food Science
Specific subject area	Food technology, Food engineering.
Type of data	Excel files (Water sorption isotherms, mid-infrared spectral data), figure (Water sorption isotherms, mid-infrared spectral data, process of acquiring spectral and sorption data).
Data collection	Water sorption isotherm data (DDI analysis), mid-infrared spectra (Attenuated Total Reflectance-Fourier Transform Infrared, ATR-FTIR).
Data source location	The experimental dataset presented in this work belongs to Centro Surcolombiano de Investigación en Café (CESURCAFÉ) from the Universidad Surcolombiana, Neiva-Huila, Colombia.
Data accessibility	Repository name: Mendeley Data Data identification number: DOI:10.17632/pffnhth7xd.2 Direct URL to data: https://data.mendeley.com/datasets/pffnhth7xd/1

## 1 Value of the Data

• We present a complete dataset of water sorption isotherms of dried parchment coffee beans processed by wet and semi-dry postharvest methods. The water sorption isotherms were determined in a wide range of water activities between 0.1 to 0.85 and a temperature range of 25, 35, and 45 °C.
• This dataset summarizes the comprehensive mid-infrared spectral data of dried parchment coffee beans processed by wet and semi-dry postharvest methods.
• The dataset can be relevant for coffee producers, researchers, and decision-makers who require fundamental knowledge to optimize the storage of dried coffee beans processed by different postharvest methods and to develop specific packaging materials/containers to maximize the quality of coffee beans in terms of sensory flavors and moisture stability.
• The dataset can be reused by other researchers to calibrate conventional sorption models for estimating both water sorption properties (monolayer moisture content, adsorption/desorption surface area, effective pore size of sorption, spreading pressure, among others) and thermodynamic properties (differential/integral enthalpy, entropy, Gibbs free energy and compensation law). It can also be used to calibrate chemometrics, data mining, and machine learning models to analyze the influence of coffee postharvest methods, water activity, and temperature on the equilibrium moisture content of coffee beans.
• The dataset allows the exploration of the effect of coffee postharvest methods on the water sorption behavior and the functional groups (fingerprint) quantified by mid-infrared spectral data.

The utility of acquiring ATR-FTIR spectra and sorption isotherms significantly benefits the scientific community. These techniques require minimal sample preparation and provide valuable insights.

Key points highlighting its usefulness include:

• **Understanding hygroscopic behavior:** the dataset provides complete water sorption isotherms for dry parchment coffee beans processed by wet and semi-dry methods. This information is crucial for understanding how different post-harvest processing methods affect the hygroscopic properties of coffee beans, which in turn influences their storage and overall quality.
• **Optimization of storage conditions:** researchers can determine the optimal storage conditions for coffee beans by analyzing water sorption isotherms. This is particularly important for maintaining sensory quality and preventing deterioration, as moisture content directly affects the stability of flavor, aroma precursor compounds, and shelf life.
• **Development of packaging solutions:** the data could be used to develop specific packaging materials and containers that maximize the quality of coffee beans. Understanding moisture sorption characteristics enables better packaging design, which can mitigate moisture-related problems during storage.
• **Calibration of sorption models:** other researchers could reuse the dataset to calibrate conventional models. This includes estimating fundamental properties such as monolayer moisture content, adequate pore size, and thermodynamic properties, which are essential for predicting the behavior of coffee beans under various environmental conditions.
• **Application of chemometrics and machine learning:** the dataset facilitates the exploration of advanced analytical techniques, including chemometrics and machine learning models. These methods can be employed to examine the impact of post-harvest processing techniques, water activity, and temperature on the equilibrium moisture content of coffee beans, thereby supporting more informed decision-making in coffee production.
• **Environmental impact considerations:** given the growing emphasis on sustainable practices, the dataset supports adopting post-harvest methods that consume less water, such as the semi-dry method. This is crucial for minimizing the environmental impact of coffee production, making the data relevant to both researchers and industry stakeholders focused on sustainability.
• **Chemical characterization:** ATR-FTIR spectroscopy enables the identification of functional groups and chemical compounds present in coffee beans. This is fundamental to understanding how different processing methods (wet and semi-dry) affect the chemical composition of beans, which in turn influences their sensory and quality properties.
• **Coffee quality analysis:** the spectral data can be correlated with coffee quality characteristics, including flavor, aroma, and stability. By identifying the compounds responsible for these properties, producers can adjust their processes to enhance the quality of the final product.
• **Monitoring transformations during processing:** ATR-FTIR spectroscopy allows for the real-time observation of chemical alterations that occur during the processing of coffee. This is beneficial for optimizing drying and storage conditions, thus ensuring the maintenance of desired quality.
• **Development of quality control methods:** the spectral data enables the development of more efficient quality control methodologies within coffee production. Producers can implement rapid analysis techniques to evaluate bean quality in real-time by establishing spectral patterns for different coffee qualities.

## 2 Background

Nowadays, adopting less water-consuming coffee postharvest methods is crucial to minimize the potential environmental impact of conventional coffee production. One method gaining particular interest is the semi-dry method, which involves the mechanical pulping of coffee beans and immediate drying, eliminating the need for large quantities of water to obtain dried parchment coffee. In contrast, the conventional wet processing method utilizes significant amounts of water. Coffee farmers and producers actively implement strategies to reduce water waste in coffee production. However, coffee obtained through the semi-dry method constitutes a heterogeneous food matrix with an additional coating on its surface (dried mucilage). This characteristic makes this type of coffee hygroscopic, behaving differently than conventionally wet-processed dried coffee beans. Thus, stakeholders in the coffee industry, including producers, researchers, and decision-makers, require specific knowledge for real-time coffee bean storage to preserve their quality. Water sorption isotherms and mid-infrared spectra are reliable tools that can help understand coffee hygroscopicity and may be suitable for optimizing storage conditions for dried coffee beans processed by wet and semi-dry postharvest methods.

## 3 Data Description

The experimental data were summarized in five Excel files, which are described below.

**Fig. 1:**
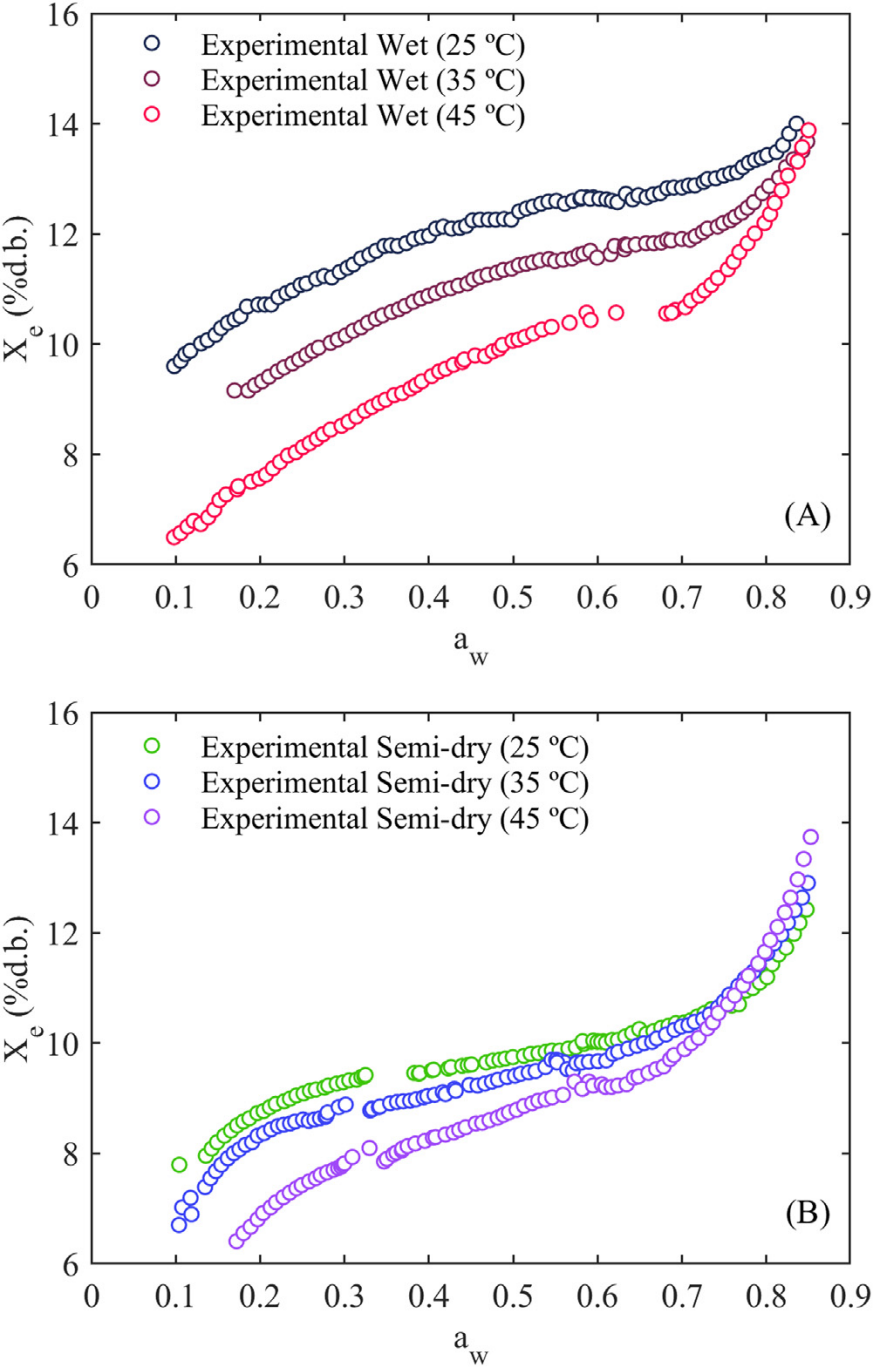
Example of the water sorption isotherms of wet-processed (A) and semi-dry processed (B) coffee beans at temperatures of 25, 35 and 45 °C.

**Experimental Water Sorption Isotherms:** This dataset compiles the water sorption isotherms of dried parchment coffee beans processed by wet and semi-dry postharvest methods. The variables in this file include water activity (ranging from 0.1 to 0.85 with a resolution of 0.01), temperature at three levels (25, 35, and 45 °C), equilibrium moisture content of coffee beans on a wet and dry basis, the zone of sorption process (adsorption 0.6 to 0.85 and desorption 0.6 to 0.1), and replicates. An example of the water sorption isotherms for both wet and semi-dry postharvest processes, specifically for the first replicate of both types of coffees at different temperatures, is shown in Fig. 1. In detail, the isotherms of both wet (Fig. 1A) and semi-dry (Fig. 1B) processed coffee beans exhibited a sigmoid-shaped ascending curve. An increase in water activity led to a rise in equilibrium moisture content. In contrast, an increase in temperature resulted in a reduction in the moisture content of coffee beans. The water sorption isotherms for both types of processed parchment coffee beans exhibited distinct behavior, unveiling differences in the influence of water activity and temperature on the equilibrium moisture content, suggesting differences in their hygroscopic behavior.

**Green Semi-dry processed_ATR-FTIR spectra:** This dataset summarizes mid-infrared spectra obtained from ground green coffee beans processed by the semi-dry method (refer to the experimental design, materials, and methods for details). The first column in this file represents the wavenumber (cm^–1^) for all infrared spectra. Columns 2 through 28 contain the absorbance values for each sample (nine samples) and their respective replicates (three replicates each).

**Green Wet-processed_ATR-FTIR spectra:** This dataset summarizes mid-infrared spectra obtained from ground green coffee beans processed using the wet method (refer to the experimental design, materials, and methods for details). The first column in this file represents the wavenumber (cm^–1^) for all infrared spectra. Columns 2 through 28 contain the absorbance values for each sample (nine samples) and their respective replicates (three replicates each).

**Parchment Semi-dry processed_ATR-FTIR spectra:** This dataset summarizes mid-infrared spectra obtained from ground coffee parchment processed using the semi-dry method (refer to the experimental design, materials, and methods for details). The first column in this file represents the wavenumber (cm^–1^) for all infrared spectra. Columns 2 through 28 contain the absorbance values for each sample (nine samples) and their respective replicates (three replicates each).

**Parchment Wet-processed_ATR-FTIR spectra:** This dataset summarizes the mid-infrared spectra of ground coffee parchment processed by the wet method (refer to the experimental design, materials, and methods sections). The first column in this file comprises the wavenumber (cm^–1^) of all infrared spectra. Columns 2 to 28 record the absorbance values for each sample (nine samples) and its replicates (three replicates each).

**Fig. 2:**
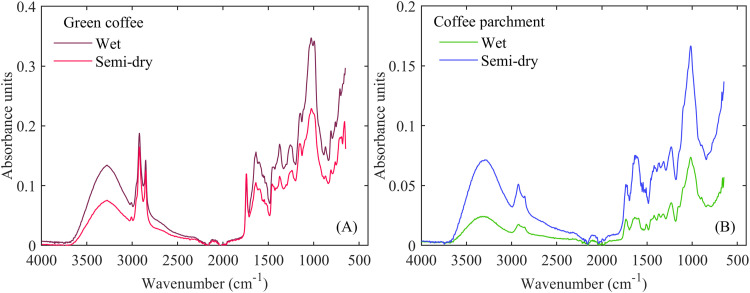
Average ATR-FTIR spectra of separated dried parchment coffee beans into ground green coffee beans (A) and ground coffee parchment (B) processed by different postharvest methods.

An average spectrum of separated dried parchment coffee beans into green coffee and coffee parchment, processed by different postharvest methods, is illustrated in Fig. 2. The mid-infrared spectra of ground green coffee beans (Fig. 2A) and ground coffee parchment (Fig. 2B), processed by different postharvest methods across the entire wavenumber range (4000 to 650 cm^–1^), exhibit well-differentiated spectral regions: (i) functional groups (3650 to 1500 cm^–1^) and (ii) fingerprint (1500 to 650 cm^–1^) [1].

The infrared fingerprints of ground green coffee beans and ground coffee parchment revealed significant differences in their absorbance units. Additionally, for the same type of ground material, the postharvest processing method resulted in a notable change in the mid-infrared spectra of the samples. The spectral behavior of the main materials that constitute the dried parchment coffee beans could provide valuable information for understanding the water sorption mechanisms. This emphasizes the potential of this dataset as a complementary approach for comprehending the water sorption process in different coffee postharvest processing methods. Furthermore, this dataset has the potential to contribute to scientific knowledge, advance research, and support real-time decision-making related to the storage of these types of coffee.

## 4 Experimental Design, Materials and Methods

Fresh coffee cherry samples (50 kg, *Coffee arabica* L.) were obtained directly from different farming areas in the Huila region of Colombia. Subsequently, the samples were processed at the Centro Surcolombiano de Investigación en Café (CESURCAFÉ). Every coffee cherry sample was divided into two batches. One batch was used to obtain wet-processed samples, and the other was used to obtain semi-dry coffee. In the former method, the cherry coffee beans were pulped in a thresher machine (Gaviota 300, Ingesec, Colombia) and fermented in plastic containers for 18 h. Subsequently, the samples were washed and sun-dried until reaching a moisture content of 10–12% (wet basis, w.b.), obtaining the wet-processed dried coffee parchment [2]. In the semi-dry method, the cherry coffee beans were pulped in a thresher machine, and then the samples were sun-dried until reaching a moisture content of 10–12% (wet basis, w.b.), obtaining semi-dry processed dried coffee parchment [3].

**Fig. 3:**
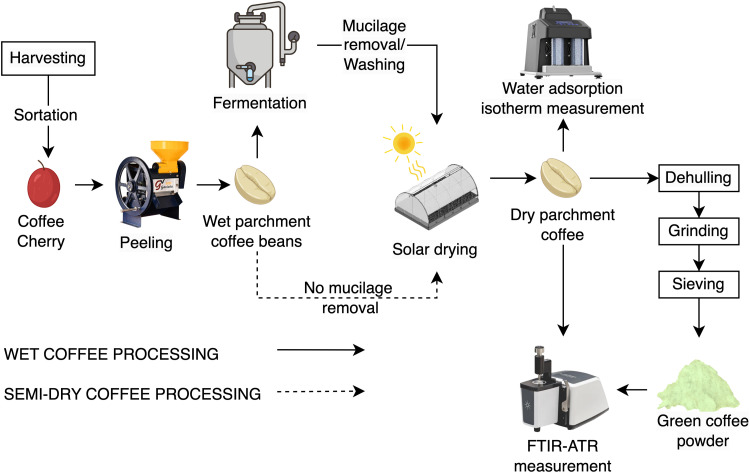
Processing procedure of coffee treatments from harvesting to obtaining the ATR-FTIR spectrum.

The samples were initially characterized by determining the moisture content of dried parchment coffee beans gravimetrically by drying 5 g samples in an oven (UF55, Memmert GmbH + Co.KG, Schwabach, Germany) at 105 ± 1 °C for approximately 24 h (until the beans reached constant weight). The water activity (a_w_) of dried parchment coffee beans was measured by placing samples of 4 g inside a vapor sorption analyzer (VSA Aqualab, Decagon Devices, Inc., Pullman, WA). The VSA instrument was previously calibrated using four saturated aqueous salt standards (13.41 molal LiCl with 0.25 ± 0.003 a_w_, 8.57 molal LiCl with 0.50 ± 0.003 a_w_, 6.0 molal NaCl with 0.76 ± 0.003 a_w_, and 2.33 molal NaCl with 0.92 ± 0.003 a_w_). The experimental water sorption isotherms were determined using the DDI method provided by the VSA instrument. Since the dried parchment coffee beans exhibited a_w_ values equal to 0.6, two sorption curves were determined. Firstly, the desorption tests were conducted in the a_w_ range of 0.6 to 0.1, and the adsorption curves were obtained in the range of 0.6 to 0.85 a_w_. All tests were performed in triplicate under an airflow of 100 mL min^–1^ and at temperatures of 25, 35, and 45 °C [4]. Furthermore, to determine the mid-infrared spectra of both wet and semi-dry processed dried parchment coffee beans, the ATR-FTIR technique was employed (Fig. 3). As the dried parchment coffee beans are mainly considered a heterogeneous material (coffee parchment which covers the green coffee beans), the samples were first dehulled using a hulling machine (ING-C-250, Ingesec, Colombia) to separate the coffee parchment peel and the green coffee beans. Further, both materials were independently ground in a milling device (Bunn G3 HD-Coffee Mill, Springfield, IL, USA). The investigation focused on fine-sized particles, defined as those retained on a standard #60 sieve with apertures of 250 μm. The separation process was conducted using a vibratory sieve shaker (EFL-2000, Endecotts LTD, London, UK) for a period of 20 min [5]. Mid-infrared spectral data were obtained using an FTIR spectrophotometer (Cary 630, Agilent Technologies, USA), equipped with a DLATGS detector and an ATR sampling attachment. Infrared spectra were recorded at wavenumbers between 4000 and 650 cm^–1^ with a resolution of 4 cm^–1^ and 16 scans. The measurements were performed in quintuplicate in a dry atmosphere and at room temperature (25 ± 0.5 °C) [6].

## Limitations

None.

## Ethics Statement

The dataset collected in this work did not involve human subjects, animal experiments, or any data collected from social media platforms.

## CRediT authorship contribution statement

**Gentil A. Collazos-Escobar:** Conceptualization, Methodology, Software, Data curation, Visualization, Writing – original draft. **Valeria Hurtado-Cortés:** Methodology, Data curation. **Andrés F. Bahamón-Monje:** Software, Data curation, Writing – original draft. **Nelson Gutiérrez-Guzmán:** Supervision, Writing – review & editing.

## Data Availability

Experimental water sorption isotherms and mid-infrared spectra of parchment coffee beans processed by wet and semi-dry postharvest methods (Original data) (Mendeley Data).
